# The Pathophysiology and Treatment of Hypertension in Patients With Cushing's Syndrome

**DOI:** 10.3389/fendo.2019.00321

**Published:** 2019-05-21

**Authors:** Mattia Barbot, Filippo Ceccato, Carla Scaroni

**Affiliations:** ^1^Endocrinology Unit, Department of Medicine DIMED, University of Padova, Padova, Italy; ^2^Department of Neurosciences (DNS), University of Padova, Padova, Italy

**Keywords:** Cushing's syndrome, hypertension, glucocorticoids, glucocorticoid and mineralocorticoid receptors Cushing's disease, cortisol lowering medications, antihypertensive therapy

## Abstract

When hypertension, a pathology that is frequently found in the general population, presents in a young patient, secondary causes such as Cushing's syndrome (CS), a rare disease characterized by long-term elevated cortisol levels, should be considered. Present in ~80% of CS patients independently of their age and sex, hypertension is one of the pathology's most prevalent, alarming features. Its severity is principally associated with the duration and intensity of elevated cortisol levels. Prompt diagnosis and rapid initiation of treatment are important for reducing/delaying the consequences of hypercortisolism. Glucocorticoid excess leads to hypertension via a variety of mechanisms including mineralocorticoid mimetic activity, alterations in peripheral and renovascular resistance, and vascular remodeling. As hypertension in CS patients is caused by cortisol excess, treating the underlying pathology generally contributes to reducing blood pressure (BP) levels, although hypertension tends to persist in approximately 30% of cured patients. Surgical removal of the pituitary tumor remains the first-line treatment for both adrenocorticotropin hormone (ACTH) dependent and independent forms of the syndrome. In light of the fact that surgery is not always successful in curing the underlying disease, it is essential that other treatments be considered and prescribed as needed. This article discusses the mechanisms involved in the pathogenesis of CS and the pros and the cons of the various antihypertensive agents that are presently available to treat these patients.

## Introduction

Cushing's syndrome (CS) is a severe clinical condition caused by prolonged glucocorticoid excess (1). While exogenous corticosteroid therapy, which is applicable to ~1% of the population, is quite common, endogenous hypercortisolism is a rare condition with an estimated incidence of 1.2–2.4 new cases/1.000.000/year (2). The syndrome is difficult to diagnose for a variety of reasons: its symptoms develop gradually, it is quite rare, and probably, most importantly, because it shares many features of a far more prevalent disorder, the metabolic syndrome. Both syndromes, are in fact characterized by abdominal obesity, glucose impairment, dyslipidemia, osteoporosis and arterial hypertension (3). Commonly featured in all forms of CS, hypertension represents the syndrome's second most common clinical finding after weight gain (4).

Hypothetically, it is more prevalent in ectopic CS (ECS) given its severity (5), although one retrospective study reported that it was more common in adrenal cases with respect to pituitary-dependent ones (CD) (6). No gender-related differences in hypertension nor marked correlations with cortisol levels have been reported (5, 7, 8). The majority of studies have shown that elevations in systolic and diastolic blood pressure (BP) values are of a similar entity in CS patients, and the loss of the typical physiological nocturnal fall, which represents an early hallmark, is almost certainly linked to a disruption in the cortisol circadian rhythm (9). Although a mild degree of overproduction of cortisol seems to have a limited impact on BP (10), prolonged excess appears to be linked to the development of hypertension due to vascular rearrangement and excessive fibrosis (11).

As pediatric CS patients tend to present hypertension even after remission, it would seem that children are prone to vascular remodeling during active disease stages, that leads to enduring hypertension even after the disease has been cured. Persistently high BP levels in these patients could also be due to the excessive glucocorticoid replacement therapy prescribed after remission (12, 13). The fact that young patients are at risk for residual hypertension and require long term monitoring to avoid post-surgery cardiovascular morbidity is well-established (14).

## Mechanisms

**Figure 1:**
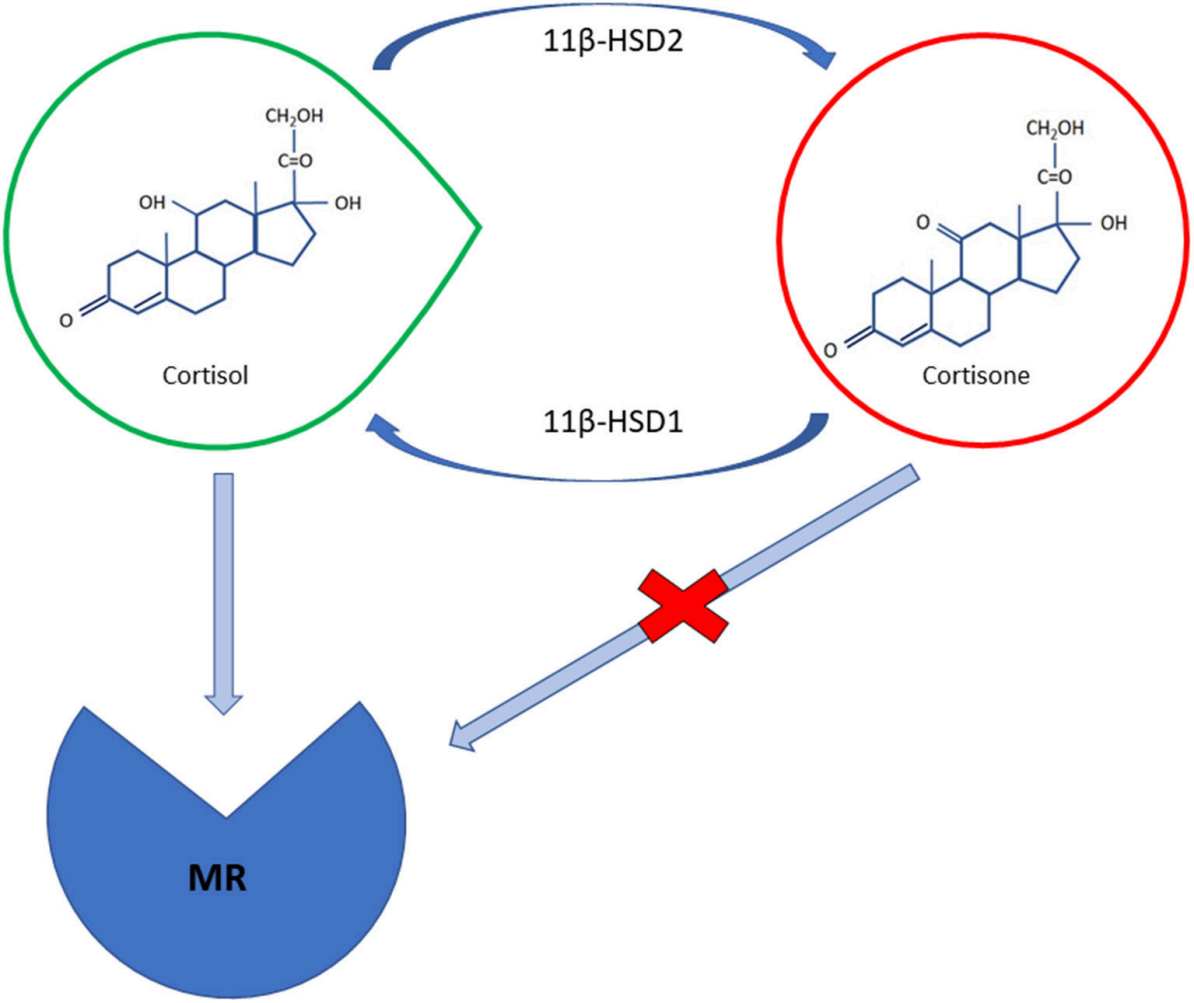
A schematic illustration of cortisol and cortisone molecular structures; cortisol can bind to mineralocorticoid receptor (MR) with the same affinity as aldosterone; the 11β-HSD2 enzyme converts cortisol in mineralocorticoid target tissues intos inactive cortisone preventing it from binding to MR. The intrinsic activity of 11β-HSD1 in peripheral tissues, especially in the liver and in adipose cells, may constitute the main determinant as far as the control of BP and the metabolic manifestations of cortisol excess are concerned.

The pathophysiology of hypertension in CS is complex. The mineralocorticoid receptor (MR) seems to be activated following saturation of the 11β-hydroxysteroid dehydrogenase type 2 (HSD2) enzyme, which converts cortisol into cortisone, thus protecting it from cortisol binding (15) (Figure 1). Glucocorticoids, which are 100- to 1,000-fold higher with respect to aldosterone, can bind both to glucocorticoid and MR. In physiological conditions, 11β-HSD2 converts cortisol to cortisone preventing it from binding to MR in target tissues, such as in the renal cortex, the colon, the salivary and sweat glands (16). Cortisol can, however, also bind to MR mimicking aldosterone action when its concentration exceeds the capacity of 11β-HSD2 to inactivate cortisol to cortisone resulting in higher sodium uptake and potassium excretion at the renal level. The blood volume expansion that follows suppresses endogenous renin secretion (17).

The molecular basis of mineralocorticoid-induced hypertension is linked to an overactivity of the epithelial Na+ channel (ENaC). It has been hypothesized that glucocorticoid receptor activation is responsible for enhanced ENaC and glomerular hyperfiltration, as neither selective mineralocorticoids nor glucocorticoid receptor antagonists appears to be able to fully revert cortisol's effects (18). These findings may explain why CS patients display more improvement when they are receiving mifepristone, a glucocorticoid receptor antagonist, than when they are taking MR antagonists (19, 20). A significant variability has also been noted as far as enzyme activity is concerned; some studies have suggested there is a positive correlation between 11β-HSD2 activity and Body Mass Index (BMI), as overweight and obese patients may have an unsuppressed renin-angiotensin system (RAS) (21, 22).

## Activation of the Renin-Angiotensin System (RAS)

Activation of the RAS via enhanced hepatic production of angiotensinogen has been described. Angiotensinogen, which is highly expressed in adipose tissues when other components of the RAS system are present, is able to generate angiotensin II and other vasoactive peptides. Low or suppressed renin levels are nevertheless quite usual in CS patients due to the negative feedback exerted by cortisol's mineralocorticoid activity, suggesting that there is a different activation mechanism (23). In fact, CS patients show a greater sensitivity to angiotensin II and its pressor activity at the peripheral levels. Glucocorticoids also enhance angiotensin II's action as a neurotransmitter leading to elevated sympathetic nerve activity, stimulating vasopressin release, and attenuating the arterial baroreceptor reflex (14).

## Effects on the Vasoregolatory Systems and Vasculature

Endothelin-1 (ET-1), a potent vasoconstrictor peptide with mitogenic and atherogenic effects on the smooth muscular cells, plays an important role in BP control in cortisol-induced hypertension. In fact, while it has been found to be significantly elevated in CS patients, a decline has been noted following treatment (24). High plasma ET-1 levels probably promote early atherosclerosis and its progression in these patients (24), although no correlation has been found with disease severity. Persistently elevated ET-1 levels may depend in some cases on residual vascular damage (25). In addition, glucocorticoids inhibit nitric oxide synthase (NOS) expression, which is essential for adequate peripheral vasodilatation and may lead to higher BP levels (26). Glucocorticoids can also impair the production of other potent vasodilatators in the vascular endothelium such as prostacyclin, prostaglandins and kallikreins (15). Prolonged hypertension and glucocorticoid exposure lead to vasculature remodeling. In fact, angiogenic and growth factors including vascular endothelial growth factor (VEGF) and insulin lead to a higher media to lumen ratio, media thickness, and wall thickness that are responsible for enhanced small artery resistance (27).

## Increased Sensitivity to Catecholamines

Cortisol is needed for the survival and maintenance of chromaffin cells, permitting them to produce epinephrine through the conversion of norepinephrine operated by the phenylethanolamine *N-*methyltransferase enzyme whose transcription is glucocorticoid-dependent (28, 29). Glucocorticoids thus modulate the synthesis of neurotransmitters and the vascular response to catecholamines; they may also contribute to the detrimental effect of cortisol on blood vessels (18).

## Metabolic Causes

As explained above, CS has many features in common with the metabolic syndrome including dyslipidaemia, insulin resistance, and impaired glucose metabolism; all are linked to visceral adiposity and associated with hypertension. As these conditions may persist even after there has been a remission in CS, they presumably contribute to maintaining, at least to some extent, high BP and the risk of cardiovascular morbidity (30, 31). In fact, symptoms of the metabolic syndrome are often noted even after CS patients have been cured (32).

Visceral obesity may contribute to the development of obstructive sleep apnea syndrome (OSAS), which, surprisingly, has at times been described in lean CS patients (33), suggesting that cortisol has a direct effect on sleep impairment (34). OSAS can exacerbate hypertension in CS by increasing sympathetic tone during hypoxemic episodes; it is also associated with insulin resistance and diabetic autonomic neuropathy (33). Regular use of continuous positive airway pressure (CPAP) therapy has been found to markedly improve BP levels in patients with severe OSAS (35).

## Cytokines and Adipokines

Visceral adiposity, which is typically present in CS and is associated with rises in pro-inflammatory cytokines such as TNF-α and IL-6, may lead to the high rates of cardiovascular morbidity observed during active disease phases (36). As some features of the metabolic syndrome may persist even after remission, the impairment in cytokine and adipokine secretion might persist in cured CS patients, contributing to a proinflammatory state and to maintaining high BP levels via enhanced sodium retention (37, 38).

## Antihypertensive Treatments

Since hypertension, which, as we have pointed out above, is quite prevalent in CS, is one of the major determinants of cardiovascular disease, it should be treated appropriately as soon as possible (1). While the underlying condition must in any case be addressed, it is important to remember that surgery is not always effective and that it may take a long time for cortisol-related comorbidities to normalize in cured patients (23). Antihypertensive treatment should be prescribed in accordance with updated guidelines both before and after surgery. Patients should also receive lifestyle education guiding them to improve their modifiable risk factors such as smoking and alcohol consumption (39). Other lifestyle changes such as losing weight and committing to an aerobic physical activity program may prove difficult to achieve in CS patients due to muscular myopathy, but they should, in any case, be encouraged (4, 40).

Almost all CS patients will require drug therapy which in most cases will involve a combination of antihypertensive agents in addition to lifestyle measures to achieve optimal BP control (23). Recently published guidelines on management of arterial hypertension have confirmed that diuretics, beta-blockers, calcium antagonists, ACE inhibitors, and sartans either as monotherapies or in combination can be used initially or at a later date to treat hypertension and to prevent cardiovascular events (39). In view of the impairment in RAS in CS, some have proposed using ACE inhibitors and sartans as the first line therapy in these cases because of their cardioprotective effects (25). It seems safe to say that since calcium antagonists have been found to be more effective than beta-blockers in delaying the progression of carotid atherosclerosis and in reducing left ventricular hypertrophy, proteinuria and stroke, they should be preferred to beta-blockers in the event add-on therapy is required (41).

The ACEinhibitor/calcium antagonist combination has proved to be more efficacious with respect to beta-blockers and diuretics in reducing cardiovascular events (42). Although beta-blockers may not represent the first-choice in CS due to their potentially unfavorable effects on glucose metabolism and heart rate, they should be taken into consideration for patients who have already experienced a myocardial infarction in which case vasodilating beta-blockers, such as labetalol, nebivolol, celiprolol, and carvedilol, should be preferred as they have fewer side effects with respect to non-selective beta-blockers (43, 44); they are also associated to a lower risk of new-onset diabetes and have fewer adverse effects on sexual function, which is often already impaired in male CS patients (4).

Should they be needed, hydrochlorothiazide diuretics can be used to manage cortisol-induced hypercalciuria to prevent calcium-containing kidney stone formation, a complication that is found in ~50% of CS cases (45, 46). Thiazides should nevertheless be used carefully in order to avoid aggravating hypokalemia, hyperuricemia, gout or diabetes, all risks factors for CS (47, 48). Caution should be used if hydrochlorothiazide therapy is prolonged as it has recently been associated with an increased risk of melanoma (49). As diuretics can reduce serum potassium levels, they too should be used with caution (23).

Spironolactone, which has been found to have beneficial effects on heart failure patients, should be used to control hypokalemia if needed (50), and it can also be used as a third-line drug to lower BP (39). As spironolactone may have anti-androgenic effects, it can be used in female patients, but it should be avoided in males since it has been associated to gynecomastia. Spironolactone metabolites such as canrenone should be preferred in male patients (51), and eplerenone can be prescribed as an alternative to spironolactone, especially in those males who have developed anti-androgen side effects (19). Although it is highly tolerated, eplerenone is not available in all countries and it is more expensive than amiloride and spironolactone (25). As no data are available on aliskiren, an expensive renin inhibitor which may cause complications in diabetics, it should be considered only when less expensive blockers of the RAS have untolerated side effects (25). Doxazosin was found to be effective by the Anglo-Scandinavian Cardiac Outcomes Trial (ASCOT) as a third-line therapy, although less efficacious than spironolactone in lowering BP in resistant hypertension (52). Although the drug has no specific contraindications in CS, it should be reserved as an add-on therapy or as a third-line option in cases of resistant hypertension after all other treatments have failed (39).

Using formulations combining more than one antihypertensive agents should be encouraged (39) as reducing the number of medications taken daily by CS patients who may need medication for numerous comorbidities (osteoporosis, diabetes, dyslipidaemia, psychiatric disorders) (4) can improve their short and in particular long-term adherence and increase BP control (53, 54). To summarize then, hypertension in CS should first be treated with ACEi or sartans at increasing doses. In the event they be unable to achieve satisfactory BP control, calcium antagonists and/or MR antagonists should be added, depending on the severity of the condition and the presence of hypokalemia (25). Acting practitioners must in any case take into consideration and evaluate both the possibility of drug interactions and any contraindications linked to hypercortisolemia (25).

## Disease Remission

As it is the only therapy that can lead to a long-term remission and reduce mortality, surgery should be attempted whenever possible for all types of CS. Surgery aims to correct hypercortisolism without creating a permanent hormone deficiency (1). When feasible, it is the first line therapy, irrespective of the lesion's site (1). Selective transsphenoidal resection of ACTH-secreting pituitary adenoma is the treatment of choice (1). When disease remission is achieved, both systolic and diastolic BP tend to improve, but approximately one-third of all adult patients continue to have systolic and three-quarters diastolic hypertension (14). Only a weak correlation has been found between the severity of baseline BP values and post-surgery hypertension. The duration of preoperative hypertension seems nevertheless to be positively correlated with its persistence after surgery and probably reflects the impact of irreversible remodeling of the vasculature caused by long-term hypertension and/or a genetic predisposition (23).

## Cortisol Lowering Medications

Drugs specific for hypercortisolism are effective in controlling BP by reducing hormonal levels and thus preventing cortisone from binding to MR receptors (Table 1). Pasireotide, the somatostatin (SST) receptor ligand, which was the first pituitary-directed drug approved for CD treatment, can bind to four out of five SST receptors and has a particular affinity for type 5 (SSTR5), the most prevalent in corticotroph tumors (55). Although pasireotide has been found to be effective in about 25% of patients by a 12-month phase III study (56), post-marketing data has shown that there was a higher rate of hormonal control in selected patients with mild CD (57–59). The drug was also found to be effective in reducing BP in CD; in fact, after a 6 month treatment period, both systolic and diastolic levels were reduced in all the treated patients, although the fall was more marked in those with controlled urinary free cortisol (UFC). The same pattern was noted at the 12 month mark, indicating an additional benefit independently of its effect on UFC secretion. Interestingly, pasireotide treatment lowered BP even in patients suffering from hypertension previously, regardless of the antihypertensive medication used (60). All the patients enrolled in a phase III trial receiving 10 or 30 mg pasireotide monthly showed a mean BP reduction of 3–5 mmHg accompanied by weight and waist circumference improvements (61).

**Table 1:** Cortisol lowering medications, their effectiveness and effects on hypertension in CS patients.

	**Drug**	**Mechanism of action**	**Dose used**	**Hormonal control**	**Effects on BP**	**Overall effect on BP**
Pituitary directed drugs	Cabergoline	Acts through D2R receptors express on adenocorticotroph	0.5–7 mg/week, oral	25–40%	↓cortisol levels ↑vasodilatation through D1 receptors	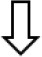
	Pasireotide	Somatostatin multi-ligand with particularly high SSTR5	300–1,800 μg/day Twice a day, sc	20–62%	↓cortisol levels	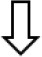
	Retinoic Acid	Reduces ACTH production through inhibition of AP-I and Nur77/Nurrl transcriptional activities	10–80 mg/day 1–3 times/day, oral	20–50%	↓cortisol levels	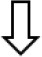
Steroidogenesis inhibitors	Metyrapone	11β-hydroxylase inhibitor	0.5–6 g/day 3–4 times/day, oral	45–100%	↓cortisol levels ↑11-deoxycorticosterone	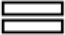
	Ketoconazole	Cholesterol side-chain cleavage complex, 17,20-lyase, 11β-hydroxylase and 17α-hydroxylase inhibitor	200–1,200 mg/day 2–3 times/day, oral	~50%	↓cortisol levels	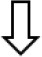
	Osilodrostat	11β-hydroxylase and aldosterone synthase inhibitor	4–60 mg/day 2 times/day, oral	~90%	↓cortisol levels ↑11-deoxycorticosterone	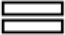
	Mitotane	Inhibition of steroid synthesis (inhibition of SOAT1, intracellular toxic lipid accumulation) + adrenolytic action	2–5 g/day 2–3 times/day, oral	~70%	↓cortisol levels ↓aldosterone levels	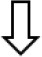
GR antagonist	Mifepristone	Glucocorticoid receptor antagonist	300–1,200 mg/day Once daily, oral	NA	↓cortisol action on GR ↑cortisol levels and its action on MR	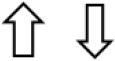

*↑ means increase; ↓ decrease; = neutral effect*.

As diabetes mellitus is a frequent adverse event, which increases the risk of cardiovascular complications, in patients taking pasireotide, BP targets should probably be lowered in these patients (39). Cabergoline, a potent dopamine agonist, was found to normalize UFC in about 30% of CD patients and to reduce BP (62–64). The improvement in hypertension could be partially attributed to the drug's relaxing effect on the vascular smooth muscles causing lower peripheral resistance (65). Originally developed as an antimycotic agent, ketoconazole has been widely used in CS because of its anti-steroidogenesis action causing inhibition of cytochrome P450 enzymes (66). Castinetti et al. reported a normalization in BP values and controlled (normalized) UFC in all the patients studied after 3–6 months of therapy with ketoconazole (67). The mean systo-diastolic BP before ketoconazole treatment was begun, i.e., 148/105 mmHg, in patients receiving and continuing to receive anti-hypertensive treatment fell to 115/85 mmHg (67). The drug's positive effects on BP were confirmed by a large retrospective multicentre study that reported that 40% of the patients studied showed an improvement in hypertension (68). Ketoconazole was also found to be superior to standard antihypertensive treatments suggesting that restoring normal cortisol levels is vital for achieving satisfactory BP control (69).

Metyrapone, another steroidogenesis inhibitor that acts by inhibiting 11-beta-hydroxylase activity, causes an increase in intermediates with mineralocorticoid activity leading to a potential worsening in hypertension and hypokalemia. These side effects are nevertheless counterbalanced by a reduction in UFC that has an overall neutralizing effect on BP (70–74). Osilodrostat, which acts on the same enzymes as metyrapone (11β-hydroxylase and aldosterone synthase), has a stronger inhibitory effect (75). Despite its effectiveness in controlling hypercortisolism and BP in a proof of concept study in CD and hypertension in primary aldosteronism (75, 76), no significant improvement was observed at the end of a 22-week phase II study (77). Combination therapy seemed at least as effective as each treatment prescribed separately (72, 78, 79); the improvement in BP was more evident when both UFC and late night salivary cortisol were normalized. There was less clinical improvement when only one of the two parameters was normalized (78, 79). BP levels, which were studied in 62 patients treated preoperatively with ketoconazole and metyrapone alone or together, were found to be lower in the controlled group with respect to the partially controlled or uncontrolled groups (72). The Metyrapone-Ketoconazole combination also produced a significant fall in systolic and diastolic BP and made it possible to reduce the number of antihypertensive drugs required in patients with severe neoplastic hypercortisolism (80).

After *in vitro* studies demonstrated their antiproliferative and proapoptotic effects on corticotroph cells, peroxisome proliferator-activated receptor-agonists (PPARγ) such as rosiglitazone or pioglitazone were utilized in CD patients because of their positive impact on insulin resistance and their anti-inflammatory, anti-oxidative, and anti-proliferative effects on the cells of the vessel walls (81–83), but their effect on ACTH and cortisol reduction in humans was found to be unsatisfactory (84–86). As some sartans such as telmisartan, irbesartan, and losartan, also have peroxisome PPAR activity, their use should probably be preferred in CS (23, 87). Mitotane, an adrenolytic agent, which is rarely used in benign CS, was found to be effective and with long-lasting effects in controlling hypercortisolism by inhibiting steroidogenesis through the impairment of mitochondrial respiratory chain activity and in toxic lipid accumulation (88–90). The fact that the agent primarily reduces diastolic values can probably be explained by the fact that low doses destroy the zona fasciculata and reticularis, sparing somehow the zona glomerulosa and its mineralocorticoid secretion (91). Retinoic acid has also been shown to exert an antiproliferative action on corticotroph cells and has anti-secretory effects by reducing proopiomelanocortin (POMC) synthesis (92). The potential of retinoic acid and its 13-cis-isomer (isotretinoin) was evaluated by two small pilot studies that examined 7 and 16 CD patients treated with increasing drug doses for 12 months (93, 94); 3/7 and 4/16 patients, respectively, were considered full responders. In addition, both studies reported an overall significant amelioration in systolic and diastolic BP during treatment (93, 94). Higher concentrations of the progesterone receptor antagonist mifepristone were able to block glucocorticoid receptors, with a binding affinity 3 times higher than that of dexamethasone without binding to the MR (95, 96). Out of the 40 hypertensive patients included in the SEISMIC study, 42.5% had a more than 5 mmHg reduction with respect to baseline values in diastolic BP after 24 weeks of therapy, and it was possible to reduce the number of antihypertensive medications in 27.5% (97). Twelve patients had worse BP control; nine showed signs of MR activation linked to ACTH and cortisol increases which may not have been completely inactivated by HSD2 in the kidney, and thus binding to the MR (98).

## Conclusions

A synergism of pathophysiological mechanisms causes the high rate of hypertension found in CS patients. The absence of nocturnal BP dipping profile is a typical feature of CS and reflects the impairment in circadian cortisol secretion. Above and beyond the hypertension that is specific to CS, a genetic predisposition could also play an important role in its development and persistence after CS remission. Controlling cortisol hypersecretion by surgical or farmacological means, such as cortisol lowering drugs or glucocorticoid receptor antagonists, can effectively lower the BP of most hypertensive CS patients and normalize it in ~50% of cases. Patients not achieving remission or presenting residual hypertension may nevertheless require a long period of time before the effects of hypercortisolism dissipate. In the meantime, they must in any case continue to assume specific antihypertensive drugs. It is important to remember in view of the fact that hypertension is such a dangerous cardiovascular risk factor, CS patients should be diagnosed and treated promptly.

## Author Contributions

MB: literature revision and drafting of the article. FC: drafting of the article. CS: critical revision of the article and final approval.

### Conflict of Interest Statement

The authors declare that the research was conducted in the absence of any commercial or financial relationships that could be construed as a potential conflict of interest.
